# Unsupervised multimodal surface registration with geometric deep learning

**DOI:** 10.1016/j.media.2025.103821

**Published:** 2025-10-03

**Authors:** Mohamed A. Suliman, Logan Z.J. Williams, Abdulah Fawaz, Emma C. Robinson

**Affiliations:** a Department of Biomedical Engineering, School of Biomedical Engineering and Imaging Science, King’s College London, London, SE1 7EH, UK; b Centre for the Developing Brain, King’s College London, London, SE1 7EH, UK

**Keywords:** Geometric deep learning, Unsupervised learning, Image registration, Cortical surface registration, Conditional random fields

## Abstract

This paper introduces GeoMorph, a novel geometric deep-learning framework designed for image registration of cortical surfaces. The registration process consists of two main steps. First, independent feature extraction is performed on each input surface using graph convolutions, generating low-dimensional feature representations that capture important cortical surface characteristics. Subsequently, features are registered in a deep-discrete manner to optimize the overlap of common structures across surfaces by learning displacements of a set of control points. To ensure smooth and biologically plausible deformations, we implement regularization through a deep conditional random field implemented with a recurrent neural network. Experimental results demonstrate that GeoMorph surpasses existing deep-learning methods by achieving improved alignment with smoother deformations. Furthermore, GeoMorph exhibits competitive performance compared to classical frameworks. Such versatility and robustness suggest strong potential for various neuroscience applications. Code is made available at https://github.com/mohamedasuliman/GeoMorph.

## 1 Introduction

The human cortex is a highly convoluted and folded structure, exhibiting intricate variations in its topography among individuals (Amunts et al., 2000; Glasser et al., 2016a). These variations pose significant challenges when aligning cortical surfaces for comparative analysis.

Cortical surface registration aims to overcome these challenges by mapping data to a global average space, where common features of brain organization overlap across individuals. Such alignment facilitates various neuroimaging analyses including investigations of cortical morphometry, functional connectivity, neurodevelopment, and neuro-surgical planning (Goubran et al., 2019; Risholm et al., 2011; Coalson et al., 2018), to mention a few. Typically, image matching is driven towards alignment of univariate summary measures of cortical folding, such as sulcal depth or average surface curvature (Fischl, 2012; Yeo et al., 2009; Robinson et al., 2014); however, more recently, as it has become clear that cortical folds poorly localize certain cortical areas, some frameworks instead target alignment of cortical areas (Nenning et al., 2017; Robinson et al., 2014, 2018; Abdollahi et al., 2014). In response to ambiguity as to what constitutes ‘optimal’ alignment of the cortex, numerous surface registration algorithms and techniques have been developed, ranging from classically-optimized methods to learning-based approaches (Heinrich and Hansen, 2020; Dalca et al., 2019); each employing different mathematical models, optimization algorithms, and similarity measures.

Conventionally, cortical surfaces are mapped to a sphere as it better captures the geodesic distances between points on the cortex. Registration is then performed by optimizing a similarity measure between the features on the target sphere and those on the deformed source sphere, while enforcing smoothness constraints. Freesurfer (Fischl et al., 1999) registers folding patterns on the surface by optimizing the mean-squared error (MSE) between a measure of average convexity across a set of subjects, and that of the individual, modulated by the inverse variance of the convexity across subjects. Spherical Demons (SD) (Yeo et al., 2009) registers two spherical images in a coarse-to-fine manner by modifying the classical diffeomorphic Demons method (Vercauteren et al., 2009), initially implemented in the Euclidean image space, using velocity vectors tangent to the sphere. Multimodal Surface Matching (MSM) (Robinson et al., 2014, (2018) also implements matching in a coarse-to-fine manner, but chooses discrete optimization over classical approaches, as this conveys flexibility with regards to choice of cost function, and greater robustness to noise and local minima. In MSM, diffeomorphisms are encouraged by imposing a biomechanically-inspired, hyper-elastic strain regularization on the deformation. Combined these features were found to significantly improve alignment of noisy areal features (resting state fMRI and T1w/T2w myelin) relative to FS, SD and volume-based approaches (Coalson et al., 2018; Glasser et al., 2016b; Robinson et al., 2014, 2018; Smith et al., 2013).

Unfortunately, as discrete optimization involves solving complex combinatorial labeling problems, this comes at the cost of relatively long run times (Section 4). Moreover, while diffeomorphisms have long been considered a pre-requisite for cortical surface registration, evidence has shown that cortical topography can vary in ways that break this assumption (Glasser et al., 2016a; Van Essen and Glasser, 2018). Resolving such problems is not straightforward, since learning how to align noisy feature maps, while breakning diffeomorphisms in a principled way, first requires better understanding of the underlying sources of variation. One potential solution to this problem is therefore to leverage learning-based image registration frameworks (Balakrishnan et al., 2019; Dalca et al., 2019; De Vos et al., 2019; Fan et al., 2018; Heinrich, 2019; Pielawski et al., 2020; Shao et al., 2021; Suliman et al., 2022), which have been shown to perform as accurately as classical approaches but with much improved efficiency. Through these, it becomes computationally feasible to align individual pairs of brains in sufficiently large numbers to map latent modes of variation (Guo et al., 2025), or generate population-average templates conditioned on demographic variables or phenotypes of interest (Dalca et al., 2019).

To date, most learning-based methods have focused on volumetric registration, building from existing well-tested and generalizable convolutional architectures, such as the U-Net (Ronneberger et al., 2015), to learn dense displacement fields. In contrast, learning for non-Euclidean mesh domains is less well established due to their lack of a global coordinate frame, which makes convolutional operations less computationally efficient and more challenging to implement (Bronstein et al., 2017; Fawaz et al., 2021). As a result, a range of different frameworks have risen up, often tailored to the demands of very different non-Euclidean domains including point-clouds (Qi et al., 2017), manifolds (Monti et al., 2017), spheres (Zhao et al., 2019) and graphs (Defferrard et al., 2016). Nevertheless, several notable learning-based cortical surface registration frameworks exist, including S3Reg (Zhao et al., 2021), which learns displacements via the implementation of Direct Neighbor (DiNe) convolutions within a spherical U-Net architecture (Zhao et al., 2019) and enforces diffeomorphisms using the scaling and squaring approach of the diffeomorphic Voxelmorph algorithm (Dalca et al., 2019); SUGAR (Ren et al., 2024) which uses a graph attention network (Velickovic et al., 2017) to learn robust surface transforms that generalize across datasets; and DDR (Suliman et al., 2022), an earlier version of the proposed framework, which employed MoNet convolutions (Monti et al., 2017) in a U-Net architecture, optimized with deep discrete registration (Heinrich, 2019). In both cases, SUGAR and DDR have been shown to learn smoother and more accurate mappings than S3Reg, most likely due to the relative lack of rotational equivariance of the DiNe convolution (Zhao et al., 2021; Ren et al., 2024; Suliman et al., 2022) when compared to MoNet (Monti et al., 2017) or graph convolutions (Velickovic et al., 2017).

For all previous methods, registration was driven using univariate summaries of cortical folding (sulcal maps and/or curvature). In this paper, we instead propose GeoMorph: a learning-based framework for robust alignment of *multimodal* cortical areal features. Since this is a much harder task due to the higher levels of noise and sparsity of fMRI and T1w/T2w myelin maps, GeoMorph again takes inspiration from MSM and DDR, to frame image registration as a deep-discrete multi-label classification problem, in which points on a low-resolution control-point grid are constrained to deform to one of a fixed, finite set of target locations, in such a way that maximizes overlap between a source (or moving) mesh and a fixed reference. However, it goes beyond the U-Net framework set out in DDR, to replace the encoder with a feature extraction network — which learns separate low-dimensional representations for each of moving and reference feature maps, to learn salient cortical features, *specific to each surface*. This is especially crucial when dealing with multiple channels of multimodal areal features, each presenting its own unique noise distribution and sparsity challenges. Smooth deformations are then enforced through use of a deep conditional random field (CRF), implemented using a recurrent neural network (RNN), which infers first-order (pairwise) regularization penalties. This process is carried out in an unsupervised way, i.e., though optimization of a similarity metric (cross-correlation) of source and target feature maps, without prior-knowledge about the desired transformation. Results show that despite its relatively weaker pairwise regularization relative to MSMAll, which implements tri-clique hyper-elastic strain terms, GeoMorph performs comparably to MSM for areal alignment of the young adult Human Connectome Project dataset (Glasser et al., 2013, 2016a); achieving this in less than 1/10000th of the run-time, while reliably generalizing to unseen data acquired with a very different scanning protocol (UK Biobank Alfaro-Almagro et al., 2018). Univariate experiments and ablation studies confirm that GeoMorph outperforms DDR and S3Reg for univariate alignment, and that all network components contribute to this boost in performance.

## 2 GeoMorph architecture

### 2.1 Background

Let **M, F** be the 3D coordinate matrices of the triangular meshes of the moving (*M*) and fixed (*F*) images, formed on a sphere 𝒮^2^, centered on the origin; each has *N*_*d*_ vertices, i.e., $M,F\in {\mathbb{R}}^{N_{d}\times 3}$. The objective of GeoMorph is to learn a spatial transformation ***Φ*** : *M* → *F* that aligns the cortical features on *M* to those on *F* in the form

(1) $$\text{Φ}={\text{ℱ}}_{\eta }(M,F)$$

upon optimizing a dissimilarity metric ℒ

(2) $$\hat{\theta }=\underset{\theta }{\text{argmin}}\;ℒ\;(\Phi_{\theta };F,M)+\text{Σ}\;(\Phi_{\theta }).$$

Here, ℱ_***η***_ (⋅) is a learnable function that is obtained using our geometric deep neural network GeoMorph, with ***η*** being the network learnable parameters. The transformation ***Φ*** is parametrized with ***θ***, while *Σ* (⋅) is a regularization function that imposes smoothness on ***Φ***. Finally, it is worth mentioning that we assume that data is presented to the network as cortical metric maps of *F* and *M*, defined on a sphere *S*^2^ that is parametrized by different resolutions (orders) of regularly sampled icospheres.

### 2.2 Method overview

**Fig. 1:**
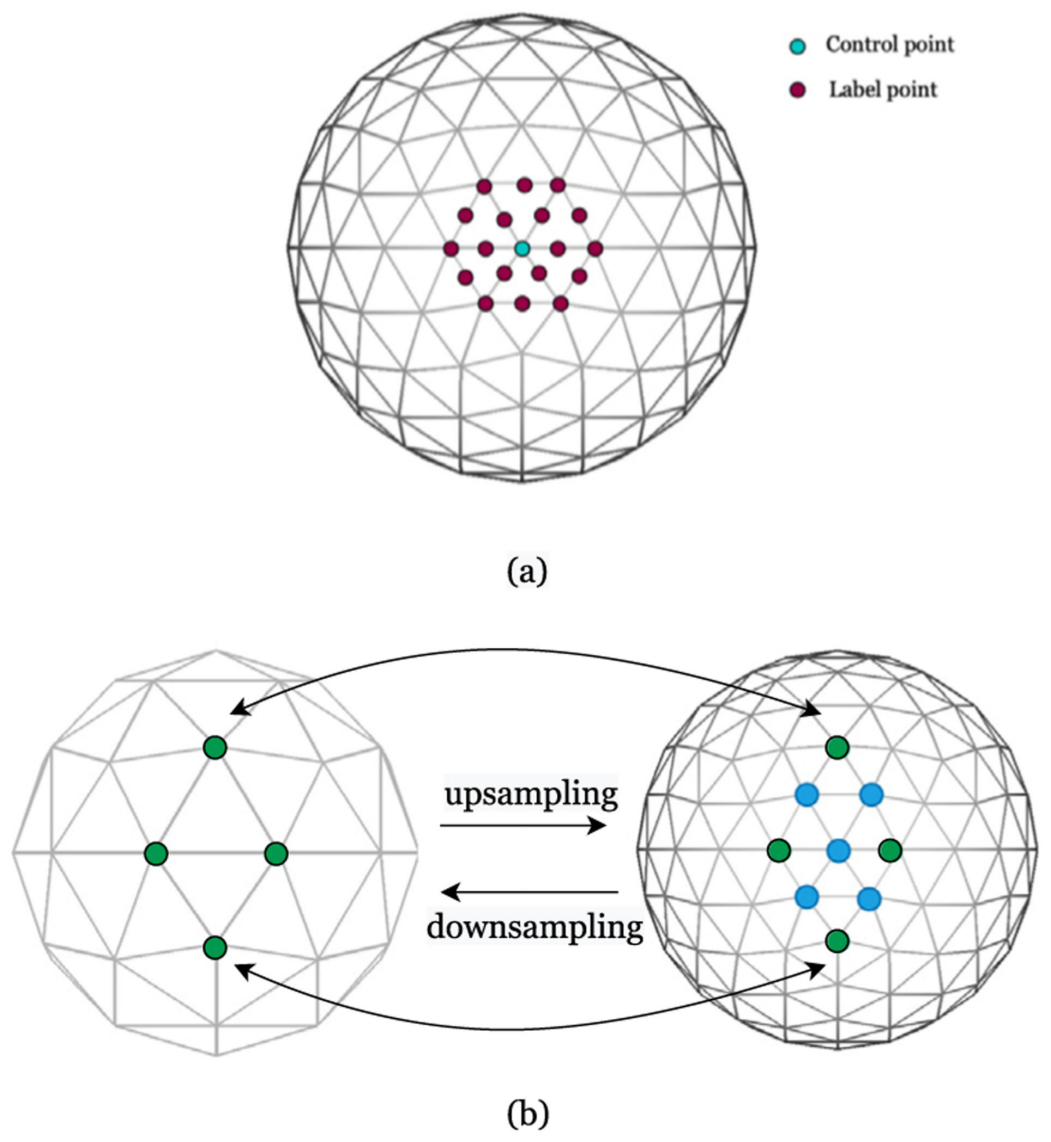
(a) Example of a control point with its labels on the surface. (b) Up and downsampling on icospheres.

**Fig. 2:**
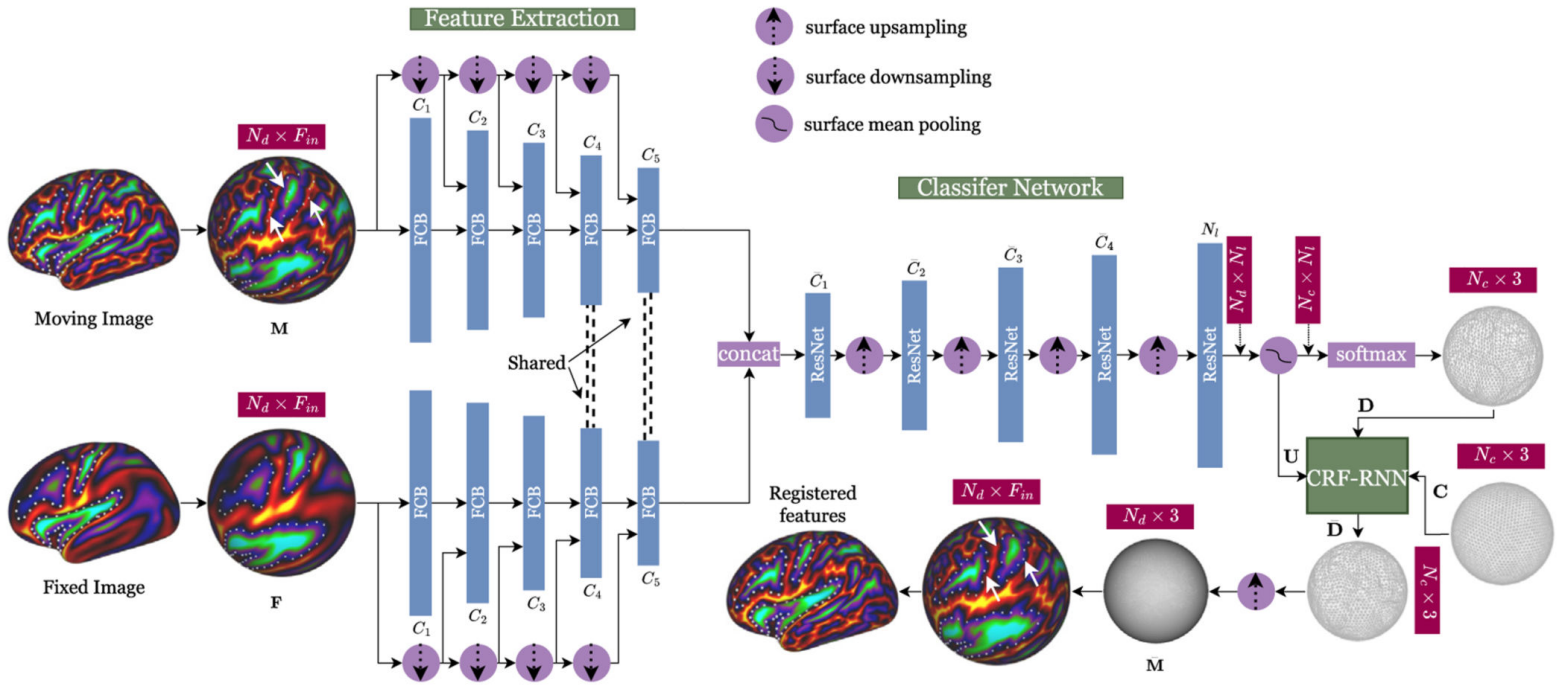
GeoMorph network architecture. The dimensions in red boxes shows the input and the output dimensions at different network stages. White dots highlight examples of reference areas within the fixed image, illustrating movements in the brain regions between moving and registered images. White arrows pointing to some areas that have moved the most.

Let ${\left\{c_{i}\right\}}_{i=1}^{N_{c}}\in {\mathbb{R}}^{N_{c}\times 3}$ be the locations of *N*_*c*_ control points on the moving sphere, generated from the vertices of a low-resolution icosphere *C* ⊂ *S*^2^ (with *N*_*c*_ ≪ *N*_*d*_), and let ${\left\{l_{i}\right\}}_{i=1}^{N_{l}}\in {\mathbb{R}}^{N_{l}\times 3}$ represent the locations of *N*_*l*_ label points, defined around each control point **c***i*, that represent all potential endpoints of the transformation ℱ_***η***_(**c**_*i*_). In all instances, the target labels are derived from the vertices of a higher-resolution icosphere (Fig. 1a). The objective of GeoMorph is, therefore, to learn the optimal label (and hence displacement) for each control point to ensure features of the fixed and moving mesh are optimally aligned. Importantly unlike classical discrete frameworks (Robinson et al., 2014, 2018), for which run-time is linked to the label dimensionality, GeoMorph is far less constrained by the extent of the label space. The general architecture of the GeoMorph network is shown in Fig. 2.

Learning on GeoMorph starts with feature extraction, which learns latent representations of features on *M* and *F* (Section 2.4). This is then followed by a classifier network which outputs $Q=\text{Softmax}(U)\in {\mathbb{R}}^{N_{c}\times N_{l}}$ softmax probabilities for each label around the control points (Section 2.5). Finally, the CRF-RNN network imposes smoothness on the learned deformation by encouraging neighboring control points to deform similarly (Section 2.6).

### 2.3 Network preliminaries

#### 2.3.1 Geometric convolutions

As input features are assumed to lie on a spherical surface, we implement surface convolutions using Gaussian mixture models, as proposed in MoNet (Monti et al., 2017) based on empirical observations that these can achieve robustness to rotational transformations (Fawaz et al., 2021).

Let *x* be a vertex on the surface (or graph) and define *y* ∈ 𝒩 (*x*) as a set of points in the neighborhood of *x*, each associated with a multi-dimensional vector of pseudo-coordinates **u** (*x, y*). Then, MoNet convolutions are defined as

(3) $$(f⋆g)(x)=\underset{j}{\sum }g_{j}D_{j}(x)f,$$

where *f* is the input feature map, *g* is a learnable filter, and *D* (*x*) *f* is a parametrizable patch operator given by

(4) $$D_{j}(x)f=\underset{y\in N(x)}{\sum }w_{j}(u(x,y))f(y),\forall j$$

which extract the values of *f* from the surface and then maps it at the neighborhood of *x* using learnable filter weights *w*_*j*_. The weights *w*_*j*_ of MoNet are formulated using the a Gaussian function in the form

(5) $$w_{j}(u)=\exp\left(-\frac{1}{2}{\left(u-\mu_{j}\right)}^{T}{\text{Σ}}_{j}^{-1}\left(u-\mu_{j}\right)\right),$$

where ***Σ***_*j*_ ∈ ℝ^*p*×*p*^ and ***μ***_*j*_ ∈ ℝ^*p*×1^ are a learnable covariance matrix and mean vector of a Gaussian kernel, respectively, whereas *p* is the dimension of the pseudo-coordinates vector **u** (*x, y*).

#### 2.3.2 Icosphere resolutions

Starting from an icosphere of order 0, which has 20 triangle faces, 30 edges, and 12 vertices, higher order resolution icospheres can be generated by hierarchically adding a new vertex to the center of each edge in each triangle (see Fig. 1b). Let the number of the vertices at the current resolution level be *N*, then, the next higher resolution level will have (*N* × 4) − 6 vertices. In contrast, the previous lower resolution level will have (*N* + 6) /4 vertices. For example, levels 1 and 2 have 42, and 162 vertices, respectively. Finally, note that with the exception of the first 12 vertices, which has only 5 neighbors, all vertices have 6 neighbors.

#### 2.3.3 Surface downsampling/upsampling

Based on the icosphere nature above, we define downsampling as the process of extracting the vertices of the lower icosphere order from the current higher order icosphere. For the upsampling, i.e., *N* → (*N* × 4)–6, we obtain the next level icosphere by inserting new vertices as the average of their direct neighbor (see Fig. 1b).

#### 2.3.4 Surface pooling

This is defined as the process of replacing each vertex and its neighbors by the mean or the max of the accumulated features from the vertex and its neighbors. Hence, we obtain a downsampled icosphere *N* → (*N* + 6) /4 with new features.

### 2.4 Feature extraction network

The primary objective of this network is to learn low-dimensional feature representations for the features on **M** and **F**, each on separate paths. The network takes the input features and the mesh topology in the form of vertex locations with neighborhood structure 𝒩, where (*i, j*) ∈ 𝒩 ⊂ { 1, …, *N*_*d*_ }^2^ indicates that vertex *i* is connected to *j* by a triangle edge. A series of feature convolutional blocks (FCBs), each taking the previous stage output and a downsampled version of the input features, are applied to learn a low-dimensional feature space from each of *M* and *F*, with only the weights of the last two FCBs being shared. This weight sharing in the last two FCBs promotes consistency in high-level feature extraction, where representations are more abstract and spatially aligned across modalities, while also reducing model complexity. In contrast, earlier layers retain independent weights to capture diverse low-level patterns that may vary significantly between input types. The feature maps from each input are then concatenated to be passed to the classifier network.

**Fig. 3:**
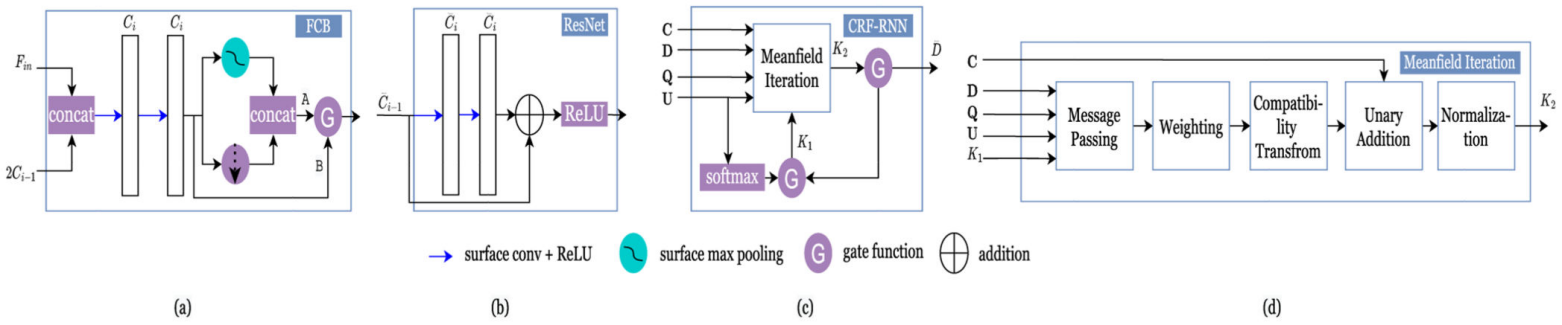
(a) FCB architecture. (b) ResNet architecture. (c) CRF-RNN architecture. (d) Meafield Iterations architecture.

At each FCB stage *i* (Fig. 3a), a total of *C*_*i*_ features are learned, using a series of two MoNet convolutional filters along with spherical polar pseudo-coordinates, mean aggregation operators, and a LeakyReLU activation with parameter 0.2. The output features are then passed through a surface max pooling operator. To allow for global feature incorporation, the max pooling output is concatenated with a down-sampled version of the LeakyReLU output. The result is then passed through a gate function *G* with *G* = *A* for *i* = 1, …, 4 and *G* = *B* for *i* = 5 (the last FCB block in the network) (see Fig. 3a).

### 2.5 Classifier network

The learned features from the previous stage are passed through a series of five ResNet-inspired blocks, each learning ${\bar{C}}_{i}$ features, with the last one learning *N*_*l*_ features. At each block, we perform two surface convolutions followed by a LeakyReLU activation with parameter 0.2 (see Fig. 3b). The output of each network is first upsampled to the next icosphere order and then passed to the next stage. The output of the final ResNet, which is of dimension *N*_*d*_ × *N*_*l*_, is regularized through downsampling to the desired control grid resolution to obtain $U\in R^{N_{c}\times N_{l}}$ (as shown in Fig. 2). The optimal label assignment is then obtained from a softmax operation on **U** to obtain **Q**. Such assignment is then combined with the labels’ spherical coordinates to deform each control point to obtain the final deformed control grid *D* ⊂ *S*^2^.

### 2.6 CRF-RNN network

On its own, the classifier is of limited use since cortical registration is an ill-posed problem with many possible solutions. Moreover, the deformation from the classifier network does not incorporate any constraint; hence, it allows each control point to move independently, which could lead to deformations with high distortions, as we will show in Section 4.

Inspired by Zheng et al. (2015) and Heinrich (2019), we introduce the CRF-RNN network in our architecture to impose smoothness by encouraging neighboring control points to deform to comparable label points. It takes as input: the control grid **C**, the deformed control grid **D**, the classifier network output **U**, and the pseudo-probabilities **Q**, to output a regularized transformation grid $\bar{D}$.

Let $Q_{\left(c_{i},l_{i}\right)}$ be the likelihood of deforming **c**_*i*_ to the label point **I**_*i*_. Moreover, we define the cost function $\varphi \left(l_{c_{i}},l_{c_{j}}\right);\varphi :S^{2}\to \mathbb{R}$, which measures the cost of deforming **c**_*i*_ and **c**_*j*_ to the label points **I**_*i*_ and **I**_*j*_, respectively. The CRF-RNN network optimizes the following CRF cost function

(6) $$E=\underset{i}{\sum }Q_{\left(c_{i},l_{i}\right)}+\underset{i\neq j}{\sum }\varphi \left(l_{c_{i}},l_{c_{j}}\right).$$

Similarly to Krähenbühl and Koltun (2011) and Zheng et al. (2015), we propose modeling $\varphi \left(l_{c_{i}},l_{c_{j}}\right)$. by

(7) $$\varphi \left(l_{c_{i}},l_{c_{j}}\right)=\mu \left(l_{i},l_{j}\right)K_{G}\left(l_{c_{i}},l_{c_{j}}\right),$$

where *μ* is a learnable label compatibility function that captures correspondences between different pairs of label points, while *K*_*G*_ is a Gaussian kernel (Krähenbühl and Koltun, 2011; Zheng et al., 2015) of the form

(8) $$\begin{array}{l} K_{G}\left(l_{c_{i}},l_{c_{j}}\right)=\\ \omega \left(c_{i},c_{j}\right)\exp\left(-\frac{1}{2\gamma^{2}}{\left(l_{c_{i}}-l_{c_{j}}\right)}^{T}\Lambda \left(l_{c_{i}}-l_{c_{j}}\right)\right). \end{array}$$

Here, *ω* are learnable filter weights, *γ* is a kernel parameter, $l_{c_{j}}$ is the new spatial location of the deformed point **c**_*i*_, while ***Λ*** is a symmetric, positive-definite, kernel characterization matrix. Note that our formulations in (7) and (8) are modified versions of those used in Krähenbühl and Koltun (2011) and Zheng et al. (2015), with a single Gaussian kernel being used in this instance, and *ω* being introduced.

To minimize (6), we use the recurrent neural network (RNN) formulation of Zheng et al. (2015) (Fig. 3c), which is based on multiple iterations of the mean-field CRF algorithm (Fig. 3d). Assuming that the CRF-RNN function is given by ${\bar{f}}_{\bar{\eta }}$, where $\bar{\eta }$ represents the network learnable parameters, and *T* is the total number of mean-field iterations, the behavior of the network can be expressed by the following set of equations:

(9) $$K_{1}(t)=\{\begin{array}{cc} \text{Softmax}(U), & t=0\\ K_{2}(t-1), & 0<t\leq T \end{array}$$

(10) $$K_{2}(t)={\bar{f}}_{\bar{\eta }}(U,C,D)$$

(11) $$Y(t)=\{\begin{array}{cc} 0, & 0\leq t<T\\ K_{2}(t), & t=T \end{array}$$

As Fig. 3d shows, the mean-field algorithm starts at the message passing stage. This applies filter *K*_*G*_ to **Q**. The weights of *K*_*G*_ are learned on the weighting stage, which can be viewed as a convolution with a 1 × 1 filter that has *N*_*c*_ input and output channels (i.e., learning *ω*). The outputs from this stage are then shared between the labels, depending on the compatibility between them, with all pairs being assigned a different penalty (i.e., learning *μ*). This operation can be performed by using a convolutional layer with a filter that has 1 × 1 receptive field and a total of *N*_*l*_ input and output channels. Next, we update **U** by subtracting the output of the compatibility stage from it. Finally, a normalization operation, using a softmax function, is performed. In all these stages, we can easily show that we can calculate error differentials with respect to the input. Thus, we can train the CRF-RNN network end-to-end utilizing the back-propagation algorithm. It is also worth mentioning that it is shown in Krähenbühl and Koltun (2011) and Zheng et al. (2015) that the mean-field iterative algorithm converges in less than 10 iterations. Once complete, the regularized deformed control grid $\bar{D}$ from the CRF-RNN network is upsampled to the input resolution using barycentric interpolation, to obtain $\bar{M}\subset S^{2}$. Features from the moving image are then resampled from $\bar{M}$ to *F* using adaptive barycentric interpolation (Glasser et al., 2013; Robinson et al., 2018), implemented through Workbench Command (Marcus et al., 2011).

### 2.7 Loss function

The network optimization is derived using an unsupervised loss function ℒ of the form:

(12) $$ℒ(\Phi ;F,\bar{M})=\lambda_{\text{sim}}{ℒ}_{\text{sim}}(F,\bar{M})+\lambda_{\text{sm}}{ℒ}_{\text{sm}}(\Phi ).$$

Here, ℒ_sim_ measures the similarity between the features on *F* and those on $\bar{M}$. We use a measure that is a sum of the MSE and cross-correlation (CC), i.e.,

(13) $${ℒ}_{\mathrm{si}m}=\frac{1}{N_{d}}\sum_{i=1}^{N_{d}}\left({\left\|F_{v_{i}}-{\bar{M}}_{v_{i}}\right\|}_{2}^{2}-\frac{\mathrm{cov}\left(F_{v_{i}},{\bar{M}}_{v_{i}}\right)}{\sigma F_{v_{i}}\sigma {\bar{M}}_{v_{i}}}\right),$$

where $F_{v_{i}},{\bar{M}}_{v_{i}}$ denote the corresponding features at vertex *i, cov* (⋅, ⋅) is the covariance operator, while *σ* is the standard deviation measure. On the other hand, the term ℒ_sm_ is introduced to allow for more user control over the balance between accurate alignment and smooth deformation and is formulated as a diffusion regularization penalty on the gradients of the ***Φ***, i.e., ℒ_sm_ = (| ▽***Φ***_**x**_ | + | ▽***Φ***_**y**_ | + | ▽***Φ***_**z**_ |, where **x, y, z** refer to the cardinal directions, while ▽ is the gradient operator. Hence, ℒ_sm_ = *Σ* from (2). To compute ▽, we apply the hexagonal filter from Zhao et al. (2019), which approximates spherical gradients on spherical surfaces (see Zhao et al. (2019, Fig. 4) for more information). Finally, *λ*_sim_ ≥ 0 and *λ*_sm_ ≥ 0 are hyperparameters.

## 3 Experiments

To validate GeoMorph, we conducted a series of experiments on real data collected as part of the adult Human Connectome Project (HCP) (Glasser et al., 2013) and the UK Biobank (UKB) (Miller et al., 2016; Alfaro-Almagro et al., 2018). In each case, *M* represents cortical features from an individual subject, whereas *F* represents features from a fixed population average atlas. We validate GeoMorph for both univariate alignment of cortical folding (sulcal depth) features, and multivariate alignment of T1w/T2w myelin maps and coarse scale surface RSNs (equivalent to MSMAll). Only left hemisphere surfaces were used.

### 3.1 Datasets

#### 3.1.1 HCP

The HCP dataset consists of cortical feature maps and meshes, derived from 1110 individuals, aged between 22 and 35 years. Participants were scanned over a two-day visit at Washington University in St. Louis, with a customized 3-Tesla Siemens Skyra, using a 32-channel head coil. Data used in this study included features derived from structural MRI and both task and resting-state functional MRI. Structural MPRAGE (T1w) and SPACE (T2w) scans were acquired with 0.7 mm isotropic acquisitions, whereas all fMRI was obtained with a resolution of 2 mm isotropic. In total, four resting state fMRI (rfMRI) scan sessions were acquired (15 min of acquisition per session), with a repetition time (TR) of 0.720s, multiband factor 8, resulting in 4800 timepoints (1200 timepoints per session per subject). A total of seven tasks were performed: emotional, gambling, language, motor, relational, social cognition, and working memory.^1^ The tfMRI were acquired after the rfMRI scans. Hence, these datasets represent entirely independent sets, allowing tfMRI to be used to robustly validate the performance of multimodal alignment for cortical areas, as demonstrated in numerous prior studies, e.g., Coalson et al. (2018), Glasser et al. (2016a) and Robinson et al. (2018).

#### 3.1.2 UKB

The UKB datasets consists of comparable features derived from 3000 UKB subjects, aged between 46 and 83 years. Scans were acquired using a 3-Tesla Siemens Skyra scanner and Siemens 32-channel head coil at 4 different locations in the UK. Structural images of 1 mm isotropic T1w and T2-FLAIR were acquired, whereas functional images were obtained at a resolution of 2.4 mm isotropic and for a total duration of 6 min. A single resting-state fMRI session was acquired, with a TR of 0.735s, multiband factor 8, resulting in 490 timepoints per subject (Miller et al., 2016; Alfaro-Almagro et al., 2018).

### 3.2 Preprocessing

In all cases, cortical surfaces were reconstructed using FreeSurfer (Fischl, 2012) following the HCP Structural Pipelines (Glasser et al., 2013; Williams et al., 2023). Cortical surfaces were extracted using both T1w and T2w images, which improves placement of the pial surface (Glasser et al., 2013). T1w/T2w maps were generated using the volumetric bias correction method described by Glasser and Van Essen (2011), with additional removal of low frequency biases in T1w/T2w across the cortical surface (Glasser et al., 2013). Resting-state fMRI was motion and distortion corrected, high-pass filtered, intensity normalized, and registered to MNI template space using FNIRT (Glasser et al., 2013; Alfaro-Almagro et al., 2018). Structured noise was removed from rfMRI timeseries using ICA-FIX (Salimi-Khorshidi et al., 2014; Griffanti et al., 2014). Cleaned timeseries were mapped to the cortical surface with ribbon-constrained volume-to-surface mapping (Glasser et al., 2013).

Task-fMRI preprocessing was the same as rfMRI. Single subject task analysis was first modeled within-run (first-level analysis), then between-runs (second-level analysis) using fixed effects general linear model with FSL FEAT (Woolrich et al., 2001). Group level task analyses were then performed using a mixed effects general linear model (Woolrich et al., 2004). Outputs were then projected to the cortical surface using ribbon-constrained volume-to-surface mapping, and minimally smoothed on the cortical surface, using a kernel of 2 mm FWHM (Glasser et al., 2013).

Given HCP and UKB data were acquired using different techniques, we first matched the histograms of the cortical features of the UKB subjects to those of the HCP subjects. Then, all features were normalized within-subject to a zero mean and a standard deviation of one, with their extreme values being clipped at ±2 standard deviations of their respective distributions. The medial wall of the cortical surface, which does not contain any cortical gray matter and represents a combination of cerebrospinal fluid, white matter and non-cortical gray matter, was considered an artifact and was masked out.

Experiments validating multimodal GeoMorph were compared against MSMAll (Robinson et al., 2018; Glasser et al., 2016b). For this cortical surface data were first coarsely aligned based on cortical folding (MSMSulc), then alignment was driven using a combination of 32 RSN spatial maps and T1w/T2w myelin. RSNs were derived from weighted dual regression of group ICA spatial maps (dimension = 40) (Glasser et al., 2016a). Cortical features were then resampled to a regular icosphere of order six (with 40,962 equally spaced vertices) using barycentric interpolation. Both myelin and functional data in HCP and UKB were smoothed on the surface using a 4 mm FWHM geodesic Gaussian smoothing kernel.

### 3.3 Implementation

GeoMorph was implemented in PyTorch, with MoNet convolutions derived from the PyTorch Geometric library (Fey and Lenssen, 2019), and the number of kernels being set to 10. In all experiments, optimization was performed using ADAM (Kingma and Ba, 2014) and the mean-field iterative algorithm in the CRF-RNN network was set to 5 iterations. Network configurations were different for unimodal and multimodal registration as follows:

#### 3.3.1 Univariate registration

Registration was driven using sulcal depth as a feature and was optimized, in a coarse-to-fine fashion, by two GeoMorph networks that were trained serially; the first of which optimizes alignment for a low resolution control point grid derived from an icosphere of order 2 (with *N*_*c*_ = 162), with *N*_*l*_ = 600 labels generated from an icosphere of order 5; the second refines alignment for a higher resolution control point grid, corresponding to an icosphere of order 4 (*N*_*c*_ = 2542), with *N*_*l*_ = 1000 label vertices that were generated from an icosphere of order 8 (which has a total of 655,362 vertices). A total of 1110 cortical surfaces with sulcal depth features from the HCP were used in this experiment. A split of 888-111-111 train-validation-test was implemented in all experiments with batch size being set to 1. The features of the FCB blocks at the feature extraction stage are set to be ${\left\{C_{i}\right\}}_{i=1}^{5}=\{32,32,64,64,128\}$ during the coarse and the fine stages with ${\left\{{\bar{C}}_{i}\right\}}_{i=1}^{5}=\{256,128,64,64,600\}$, *γ* = 0.2, and *λ*_sm_ = 0.6 during the coarse stage and ${\left\{{\bar{C}}_{i}\right\}}_{i=1}^{5}=\{256,128,128,128,1000\}$, *γ* = 0.2, and *λ*_sm_ = 0.6 during the fine stage. Finally, we set *λ*_sim_ = 1 for both stages. The learning rate was set to 10^−3^ and the model was trained by minimizing the function in (12). The coarse network was trained for 100 epochs, each time learning the deformation at the control grid level, upsampling it to the input resolution level using barycentric interpolation, and then resampling the input sulc features to this newly deformed sphere to compare with the fixed image sulc features. Once training was done, the network parameters that provided the best validation score were saved. The resulted deformed moving image was then passed to the fine stage, and this new network was trained for 100 epochs. The final performance on the test set was reported using network parameters that provided the best validation score.

#### 3.3.2 Multimodal registration

Training was performed using myelin and RSNs derived from both HCP and UKB, with the learning rate being set to 2*e*^−4^. The batch size was set to 1 for all experiments and train-validation-test splits of 801-100-100 and 2556-200-200 were used for the HCP and UKB respectively. The HCP and UKB data were stacked together and then randomly shuffled during the training and the validation phase. Unlike univariate registration, no further improvement in performance was observed for multi-stage image registration; hence, a single network with high resolution control point grid was used, set to the resolution of an order 4 icosphere i.e., *N*_*c*_ = 2542, with *N*_*l*_ = 600 lying on an icosphere of order 6. Moreover, we let ${\left\{C_{i}\right\}}_{i=1}^{5}=\{32,32,64,64,128\}$, *γ* = 0.2, *λ*_sim_ = 1, *λ*_sm_ = 0.6, and we set ${\left\{{\bar{C}}_{i}\right\}}_{i=1}^{5}=\{256,128,128,128,600\}$. Training was carried out in two stages. First, the network was pretrained using an autoencoder whose architecture mirrored that of the feature extraction network — with an identical encoder and equivalent decoder layers implemented in reverse. Following that, the GeoMorph network was trained for 100 epochs and the network performance with the best validation score was reported. A discussion on the impact of the various parameter sets mentioned above can be found in Section 4.3.

### 3.4 Benchmark methods

GeoMorph was benchmarked against SD, MSM, Freesurfer, and the learning-based method S3Reg. The validation was performed using the official implementations of SD,^2^ MSM Pair,^3^ MSM Strain,^4^ and S3Reg.^5^

#### 3.4.1 Univariate registration

To achieve fair comparison across all methods, the hyper-parameters were tuned for each, and performance across all parameter configurations were reported. The following parameters were optimized:

- SD: The number of smoothing iterations used to smooth the final displacement field (in the Spherical Demons second step) was selected from [1, 5, 10], whereas the smoothing variance *σ*_*x*_ was varied over [1, 2, 6, 10] (hence, ending with a total of 11 experiments).
- MSM: Two versions of MSM were used: MSM Pair (Robinson et al., 2014), which uses first-order (pairwise) penalties, and MSM Strain (Robinson et al., 2018), which applies high-order smoothness constraints derived from physically relevant equations of strain energy. A single regularization parameter was set at the 4 stages of the registration (coarse to fine). Here the regularization was varied over 22 weighting factors that differed across MSM Pair (*λ* ∈ [0.0001, 0.2]) and MSM Strain configurations (*λ* ∈ [0.0001, 0.9]).
- S3Reg: The network employs distinct regularization parameters at each of its 4 registration stages. To achieve optimal performance, 7 experiments were conducted, each utilizing specific configurations of regularization penalties for the corresponding sets: (refer to Zhao et al. 2021 for more information): [2, 5, 6, 8], [2, 10, 12, 20], [2, 10, 12, 14], [2, 5, 12, 16], [2, 10, 6, 8], [2, 10, 12, 8], and [2, 5, 6, 16]. A fundamental limitation of S3Reg is that the hexagonal filter implemented in Zhao et al. (2019) is not rotationally equivariant due to the lack of a global spherical coordinate system; hence, it flips directions at the poles and generates distortions. S3Reg overcomes this by using a combination of three networks, each trained on a different rotated version of the input. In each case, S3Reg networks were trained for 100 epochs at each registration level, and the performance of the network with the best validation score was reported.
- Freesurfer: The method is not tunable, and therefore results were reported for its default parameterization.

Note that all these frameworks perform coarse-to-fine, multi-stage registration over 4 icosphere resolutions. Moreover, S3Reg framework has an additional spherical transform network that seeks to enforce a diffeomorphic registration.

#### 3.4.2 Multimodal registration

Multimodal experiments benchmark GeoMorph solely against MSM — as the most highly optimized and rigorously benchmarked classical framework for multimodal image registration. In this comparison, two variants of MSM were used: namely MSMSulc and MSMAll, as outlined in Section 3.2. Moreover, two versions of GeoMorph were presented: GeoMorphSulc which denotes alignments achieved using GeoMorph driven by sulcal depth features, and GeoMorphAll which corresponds to alignments obtained utilizing myelin and rfMRI features from both HCP and the UKB datasets. The inclusion of GeoMorphSulc aims to showcase the improvements attained through multimodal registration.

### 3.5 Evaluation measures

The performance of all methods was compared based on their alignment quality, assessing how well features in the source and target meshes overlap using cross-correlation (CC) similarity, and also on the smoothness of the resulted deformation using areal and shape distortions. The distortions are calculated from the local deformation (**F**_*pqr*_) of each triangular face, defined by vertices **p, q, r**. The eigenvalues of **F** (*λ*_1_ and *λ*_2_) represent principal in-plane stretches (Knutsen et al., 2010), such that relative change in the area may be described by *J* = *λ*_1_/*λ*_2_, whereas the relative change in shape may be described by *R* = *λ*_1_/*λ*_2_.

The areal distortion is defined as log_2_ *J* = log_2_ (Area_1_/Area_2_), while shape distortion is defined by log_2_ *R*. Each of these measures was measured across all registered surfaces

Multimodal registration derived with myelin and rfMRI was also evaluated using HCP tfMRI data. In this case, improvements in alignment were assessed qualitatively and quantitatively upon comparing the group mean activation maps using a ‘cluster mass’ measure (Robinson et al., 2014; Glasser et al., 2016a); this quantifies the size of the supra-threshold clusters and the magnitude of the statistical values within them, and is obtained using the following formula: *CM* = ∑ _*i*∈ 𝒯_‖*z* (**x**_*i*_) ‖*A* (**x**_*i*_). Here, **x**_*i*_ is the vertex coordinate, *z* (**x**_*i*_) is the statistical value at **x**_*i*_, *A* (**x**_*i*_) is one third of the area associated with **x**_*i*_ (Winkler et al., 2012), and 𝒯 represents the set of vertices with | *z* (**x**_***i***_ **)**| ≥ 5. The area *A* **x** is obtained from a share of the area of each mesh triangle connected to it in the mid-thickness surface. The cluster mass was obtained for each contrast within the set of 7 HCP task experiments, a total of 86 contrasts. Note that a higher cluster mass measure indicates a better registration performance.

## 4 Results

### 4.1 Univariate registration

**Fig. 4:**
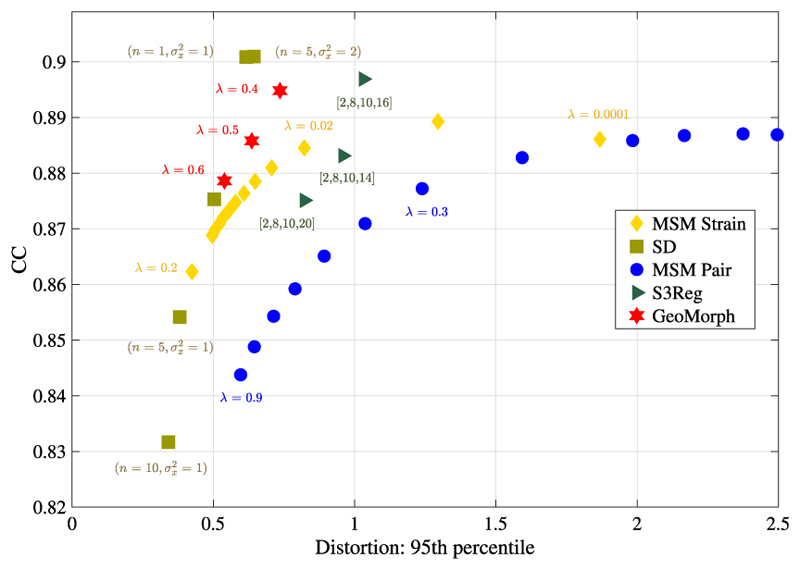
Similarity performances of all methods vs. the 95th percentile of the areal distortion at multiple regularization levels across runs.

Fig. 4 illustrates the similarity performances of various runs of all methods plotted against the 95th percentile of the absolute value of the areal distortion. For each similarity level, GeoMorph exhibits distortions falling within the range of the best classical methods (SD and MSM Strain) and demonstrates reduced extremes of areal distortions compared to S3Reg. Table 1 summarizes the performance of all surface registration frameworks on the task of sulcal depth alignment. In each case, results are reported for the configuration that generated a mean CC value of approximately 0.88 (as this is the best CC value that all methods can achieve). Since the P-values of all methods are very close and they are all above the threshold for statistical significance (i.e., 0.05), performance should, therefore, be judged in terms of which methods achieve the lowest mean, maximum, 95th percentile, and 98th percentile values of the distortion. In this, GeoMorph is the second best performing framework (behind SD but better than MSM Strain). On the other hand, S3Reg and MSM Pair exhibit much poorer performance. It is worth noting that these values represent the optimal performance of S3Reg, across all runs. Moreover, it is worth mentioning that, with the exception of S3Reg, all methods do not have a non-positive Jacobian determinant, indicating that they do not produce self-intersecting vertices (i.e., diffeomorphic deformation). In terms of average run time, when utilizing a PC equipped with an NVIDIA Titan RTX 24 GB GPU and an Intel Core i9-9820X 3.30 GHz CPU, GeoMorph exhibits the least GPU and CPU times among all methods.

**Fig. 5:**
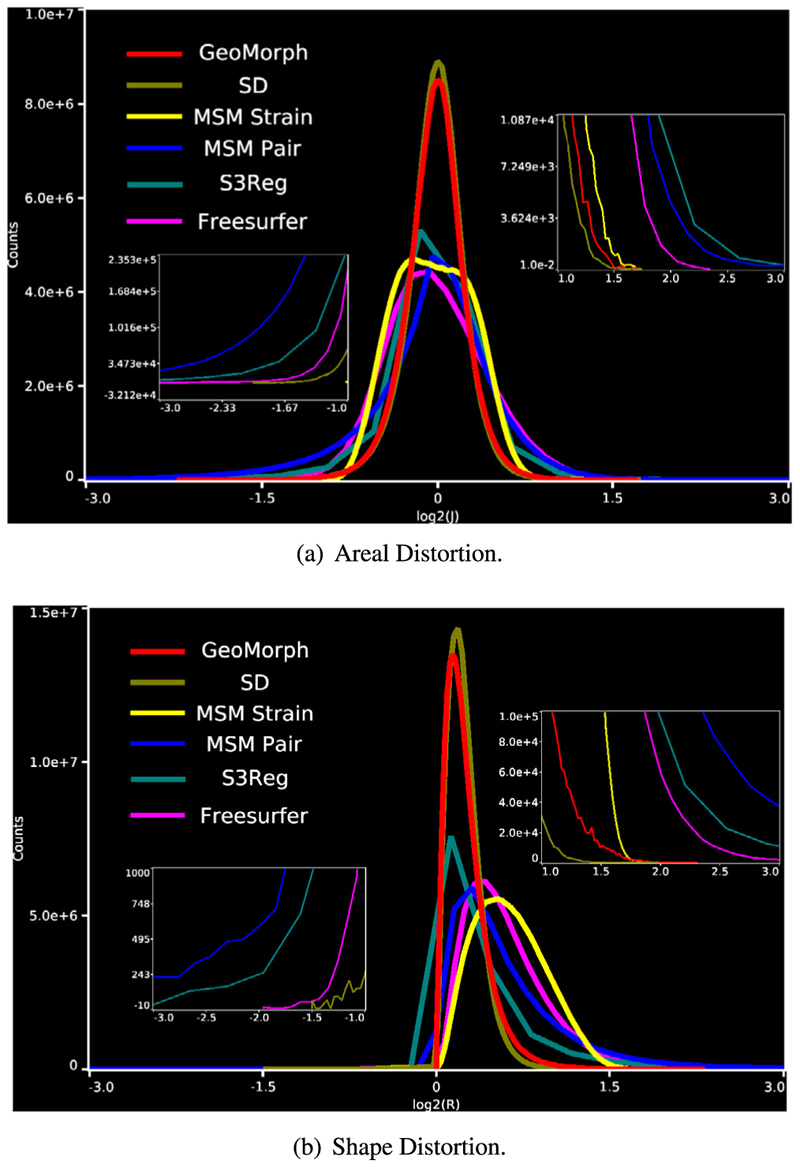
Histogram plots comparing areal and shape distortions across all test subjects.

In Fig. 5, we present histograms showcasing the distribution of both areal and shape distortions, across all test subjects, at CC of 0.88. By analyzing Fig. 5a, we observe that SD and GeoMorph predominantly exhibit areal distortions centered around zero. On the contrary, MSM Pair, S3Reg, and (to a lesser degree) Freesurfer demonstrate pronounced extreme distortions across subjects, as evidenced by the presence of long tails in the histogram. This trend is also observable in Fig. 5b, where we observe that the distribution of shape distortions, for SD and GeoMorph, predominantly falls below one. Conversely, other methods exhibit significant instances of extreme distortions.

**Fig. 6:**
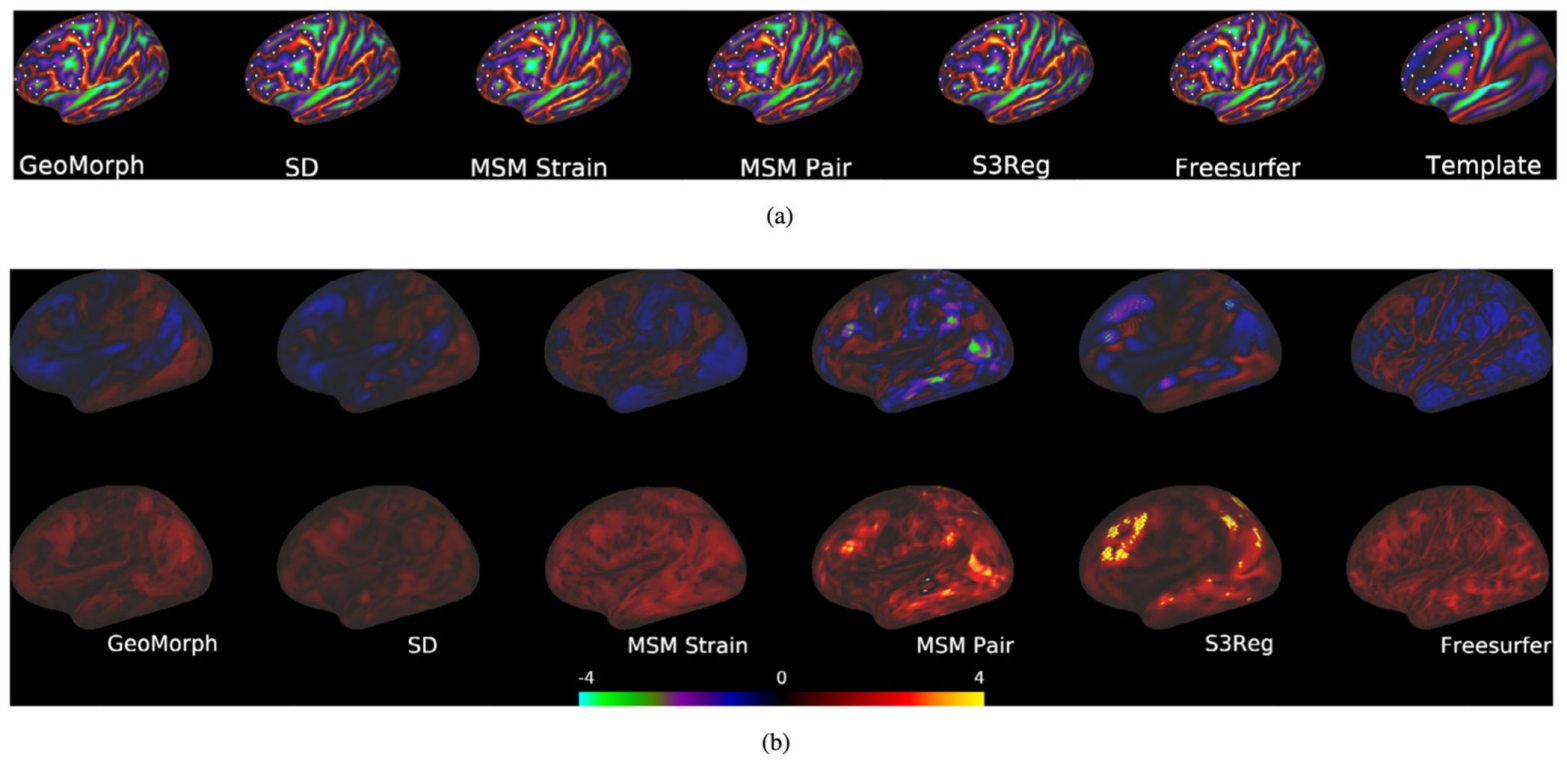
(a) Registration Performance. (b) Areal (top) and shape (bottom) distortions

In Fig. 6, we visually evaluate alignment quality, for all bench-marked methods, on one subject from the HCP dataset that exhibits atypical cortical folding patterns. Additionally, we examine the areal and shape distortions resulting from these methods. The figure illustrates that GeoMorph, SD, and MSM Strain achieve favorable alignment, with minimal distortions. In contrast, the alignments produced by MSM Pair and S3Reg are characterized by regions with very high distortions.

### 4.2 Multimodal registration

**Fig. 7:**
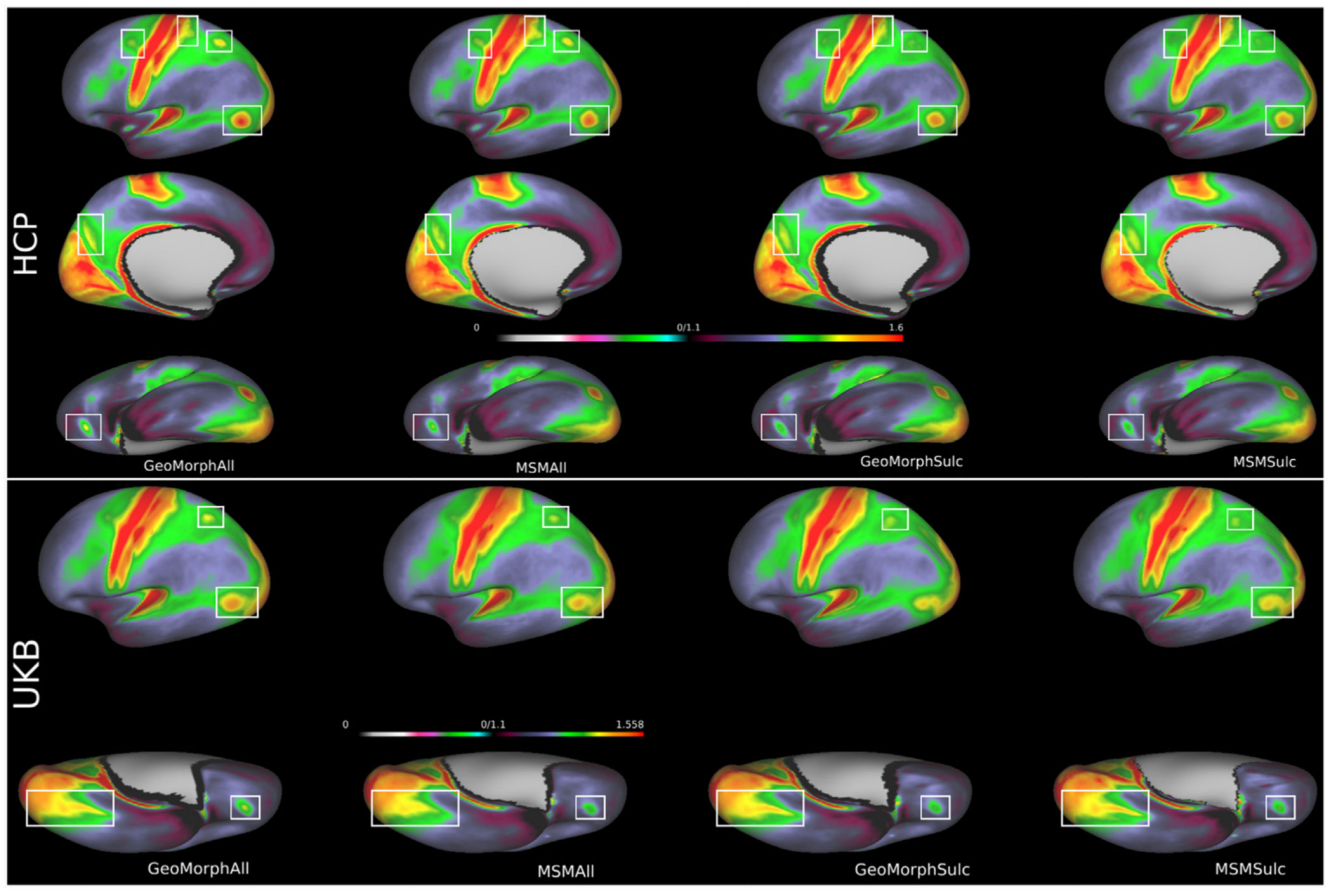
Intersubject averages of myelin features in HCP (top) and UKB (bottom). Regions in the brain that exhibit significant disparities in contrasts and enhanced performance between these methods are denoted by the areas highlighted within white boxes.

Fig. 7 illustrates the sharpness of cross-subject T1w/T2w myelin map averages for all methods, benchmarked for both HCP and UKB datasets. Areas of distinct improvement, following multimodal alignment, are highlighted by white boxes. The results show that GeoMorphAll yields a sharper and clearer average map, relative to MSMAll. Improvements are particularly distinct for the UKB dataset; this might be because MSM was originally optimized for the HCP.

**Fig. 8:**
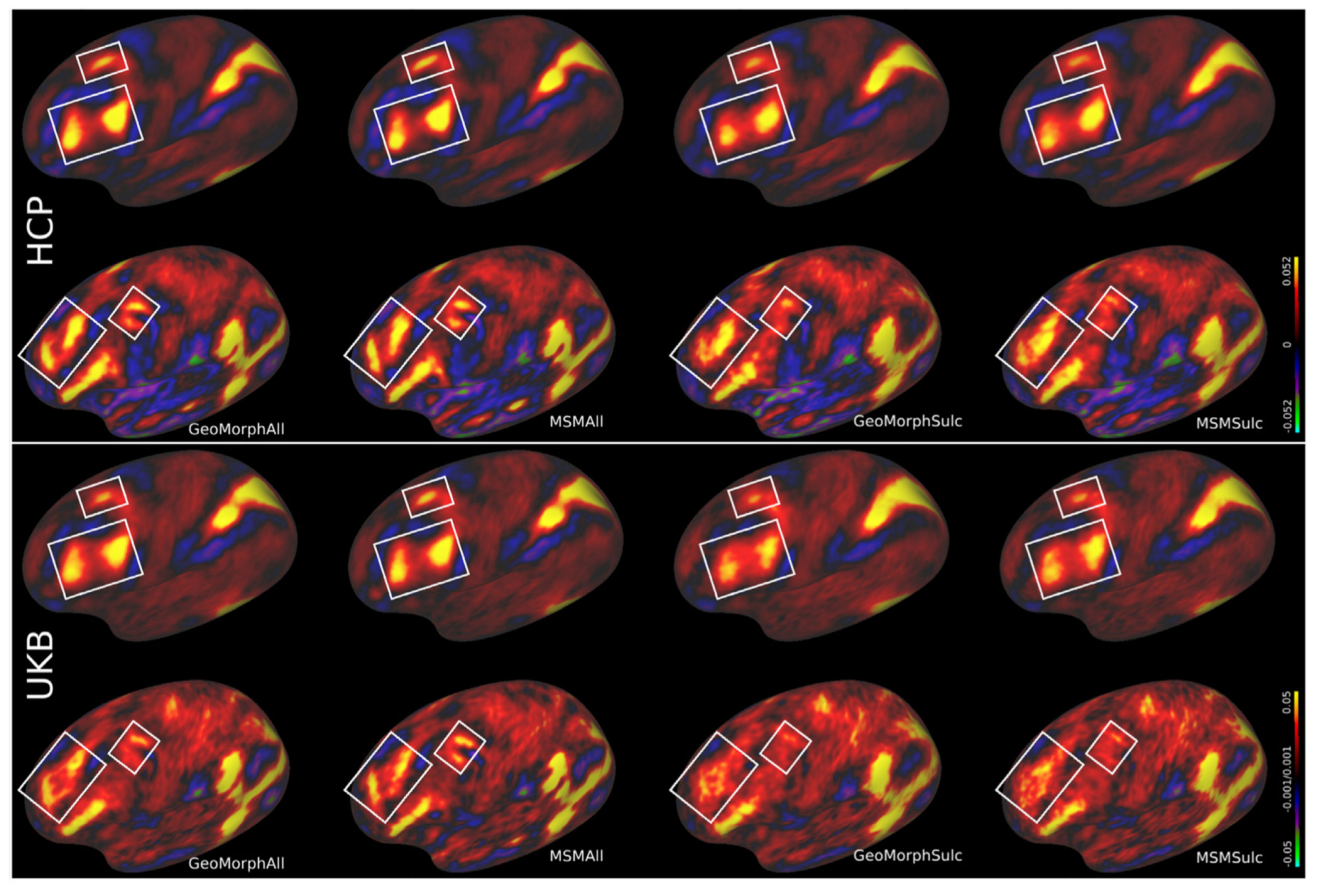
Intersubject averages of rfMRI features in HCP (top) and UKB (bottom). Regions in the brain that exhibit significant disparities in contrasts and enhanced performance between these methods are denoted by the areas highlighted within white boxes.

Similar patterns of results are shown for the cross-subject averages of resting state spatial maps (Fig. 8). In this case performance relative to MSM is more variable across the cortex; however, the improvements of GeoMorphAll and MSMAll, relative to GeoMorphSulc and MSMSulc, remain clear. Note that GeoMorphSulc is the same unimodal (sulcal depth) GeoMorph used in Section 4.1, renamed here for consistency with MSM terminology.

Table 2 provides a quantitative analysis of the registration performance, on myelin and rfMRI, in terms of CC similarity and distortion statistics. The results reveal that GeoMorphAll demonstrates comparable CC performance to MSMAll, across both datasets. Notably, while MSMSulc exhibits superior distortion measures, this is to be expected as it reflects a highly constrained folding based alignment. It is clear this comes at the expense of much poorer CC performance (Figs. 7 and 8).

**Fig. 9:**
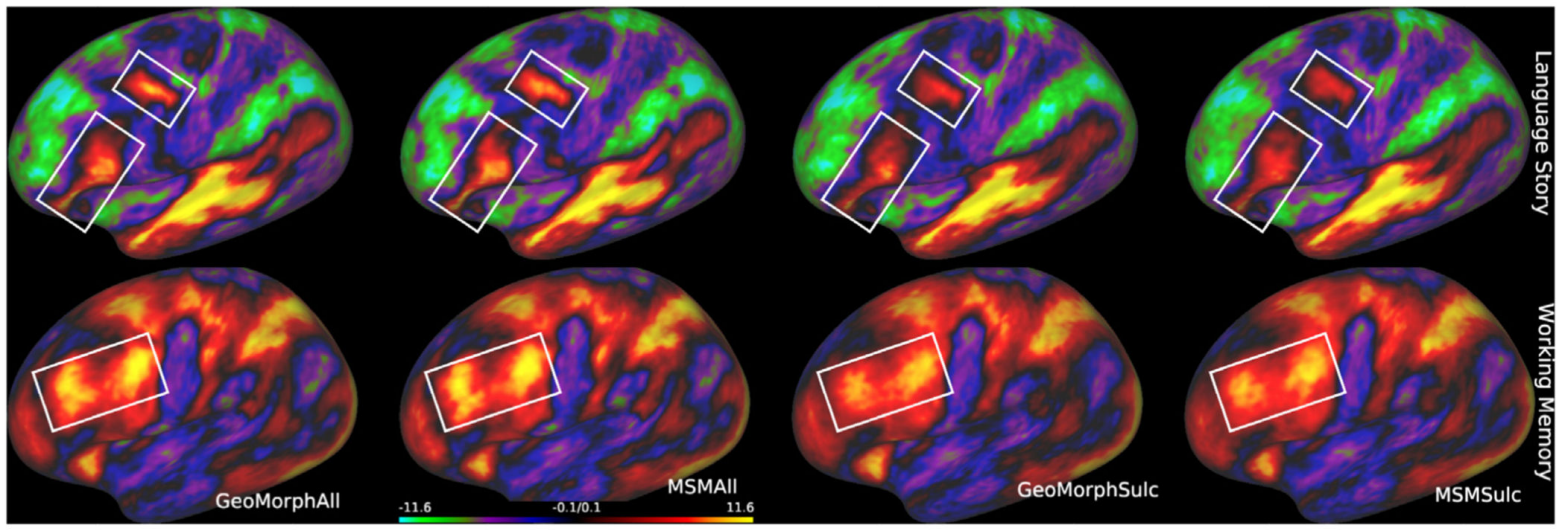
Comparison of group Z-statistic spatial maps of all methods for (a) Language Story task. (b) Working Memory task. White boxes highlight improvements in the sharpness of the contrast, with GeoMorphAll and MSMAll maintaining a close comparable performance that outperforms unimodal methods.

Validation on independently acquired tfMRI is reported in Table 3. Results reproduce previous findings from MSM studies (Coalson et al., 2018; Glasser et al., 2016b,a; Robinson et al., 2014, 2018; Smith et al., 2013) that suggest that multimodal alignment significantly improves the overlap of cortical functional areas across subjects, as evidenced by improvements to the extent and peak z-values of group task statistics (as summarized by the cluster mass measure). Overall performance of GeoMorphAll is comparable to that of MSMAll. Fig. 9 presents visual results for the working memory and language story task. The figure shows the improvement in the sharpness of the contrast in multiple areas within the brain for both GeoMorphAll and MSMAll, both outperforming unimodal alignment methods using sulcal depth.

### 4.3 Ablation and hyperparameter tuning of multimodal alignment

**Fig. 10:**
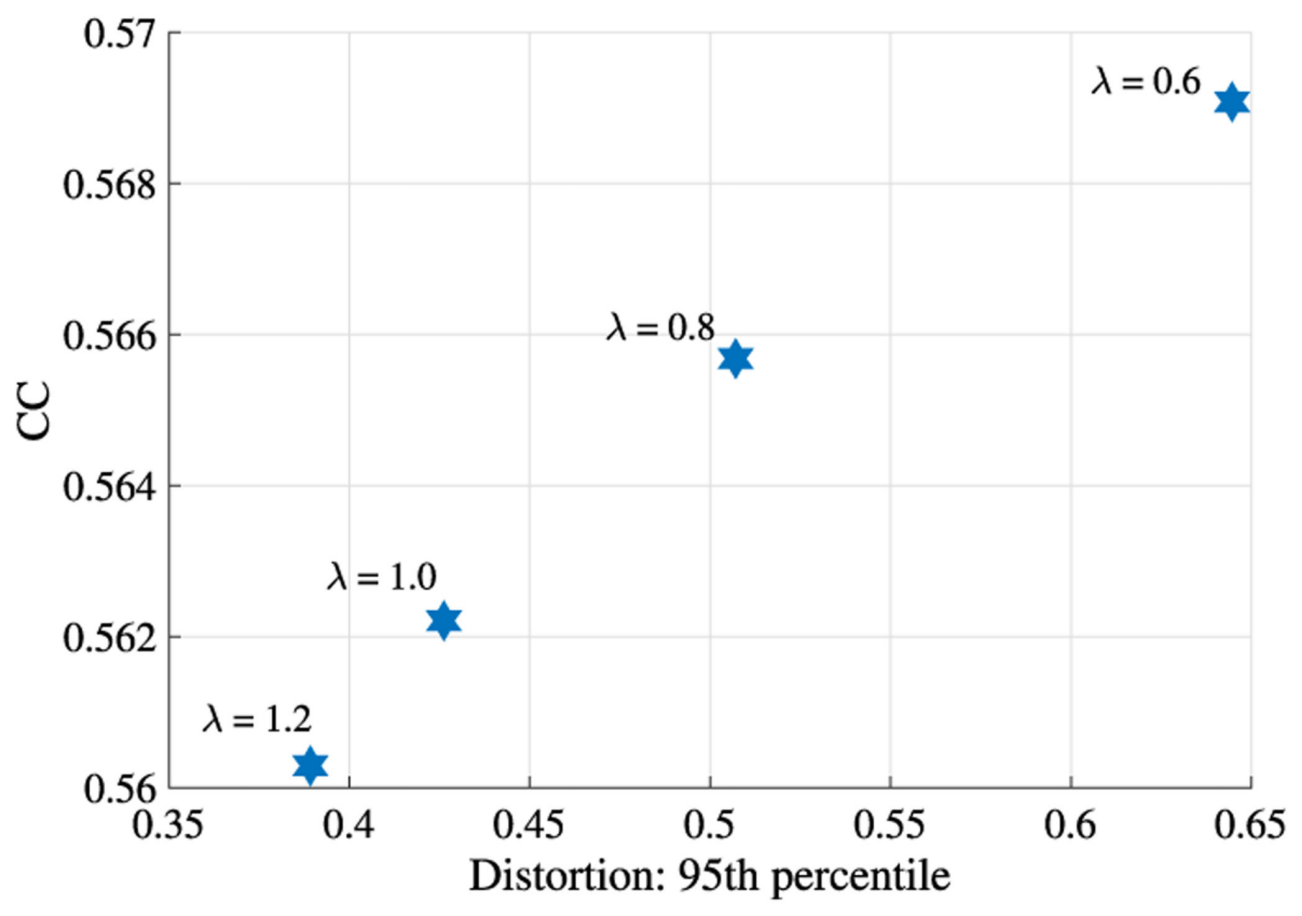
Similarity performances vs. the 95th percentile of the areal distortion at multiple regularization levels.

Fig. 10, reports performance of the network as the regularization parameter *λ* is changed, indicating an inverse relationship between image similarity and distortion. The impact of control point grid resolution is reported in Table 4. These results demonstrate that as the resolution of the control grid increases, the model’s performance improves. Table 4 also shows the model performance on HCP with label points lying on an icosphere of order 5. The results indicate a slight reduction in similarity performance compared to the icosphere of order 6 (as shown in Table 2), along with higher peak distortions.

Ablation analyses, investigating the benefits of different components of the network are provided in Table 5. These demonstrate that integration of a CRF-RNN network, reduces distortion (across all measures) whilst not impacting the goodness of alignment (as quantified through the CC similarity measure). On the other hand, to show that a discrete-based approach combined with the CRF-RNN is capable of learning larger and smoother deformations, we compare the results on the HCP in Table 5 using our method versus a method where deformations are learned as a continuous velocity field with scaling and squaring layers used instead of the CRF-RNN to ensure smoothness. The scaling and squaring layers implement the exponentiation of the mapping as described initially in Ashburner (2007) and later generalized to learning-based frameworks by Dalca et al. (2018). In this experiment, the final layer of our classifier network is replaced with a layer that outputs a continuous velocity field, which is then added to the control points. Additionally, the CRF-RNN network is replaced with a set of six scaling and squaring layers operating on the spherical space, as implemented by Zhao et al. (2021) based on the theories of Yeo et al. (2009). Based on the results in Table 5, it is evident that GeoMorphAll with CRF-RNN imposition can learn larger and smoother deformations, resulting in better alignment of the subjects.

Next, we investigate the impact of the feature extraction network. For that, we employ the settings from the univariate registration problem, and we use the HCP sulcal depth feature. Instead of following the architecture in Fig. 2, we concatenate the fixed and moving images at the input and pass them through a single-path feature extraction pipeline as introduced in DDR (Suliman et al., 2022). We present the results of this experiment in Table 5 for the configuration that achieved a mean CC value of approximately 0.875 for a fairer comparison. The results indicate that including the feature extraction network improves distortion measures across all metrics.

Finally, to validate the generalization of the framework, we trained the network on the HCP dataset only and then validated the performance using the UKB dataset. The results in Table 6 show a minor hit in the performance, confirming that our model generalizes well.

## 5 Conclusions and discussions

In this paper, we presented GeoMorph, an innovative geometric deep-learning framework specifically designed for multimodal cortical surface registration. GeoMorph aims to learn a smooth displacement field that effectively aligns the features on the moving surface with those on the target surface. By leveraging independent feature extraction and deep-discrete registration, GeoMorph captures crucial characteristics of cortical surfaces and optimizes feature overlap to achieve improved alignment. To ensure the generation of visually coherent and anatomically plausible deformations, we incorporate a regularization network based on a deep conditional random field. Experimental results using sulcal depth features demonstrate that GeoMorph outperforms existing deep-learning methods by achieving enhanced alignment and generating smoother deformations. Moreover, GeoMorph exhibits competitive performance on multimodal alignment, when compared to classical frameworks, while demonstrating vastly reduced run times. This computational efficiency should prove beneficial for large open data sets or clinical applications, especially when a pre-existing cortical areal template does not exist; this is particularly important in the context that multimodal templates capture crucial attribute-related trends, which sets GeoMorph apart from conventional methods that only generate templates that capture dominant folding patterns (Dalca et al., 2019).

When comparing the performance of GeoMorph against the deep-learning based method S3Reg (as shown in Table 1), it becomes evident that S3Reg exhibits significant high peak distortions. This issue is likely attributed to the hexagonal filter utilized in S3Reg, which lacks robustness to rotational transformations due to the absence of a global spherical coordinate system. The solution proposed by S3Reg, which involves a combination of three networks, does not seem to fully overcome this problem, despite efforts to enforce diffeomorphisms through using the scaling and squaring approach from the diffeomorphic Vox-elmorph algorithm (Dalca et al., 2019). Contrastingly, the GeoMorph structure does not explicitly enforce diffeomorphisms. Nevertheless, all yielded results are discovered to be diffeomorphic—a foreseeable outcome due to the strong regularization imposed by the GRF-RNN network.

A future expansion of GeoMorph aims to explore relaxing this requirement within the framework. The motivation for delving into this aspect arises from recognizing that achieving perfect alignment on functional topographies proves unattainable with diffeomorphically-constrained deformations. This observation is evident in 10% of subjects for area 55b in Glasser et al. (2016a), as underscored in Schneider et al. (2019). Conventional diffeomorphic registration approaches, as showcased in Thual et al. (2022), tend to exhibit reduced performance in such scenarios. Finally, it should be highlighted that while MoNet has empirically demonstrated robustness to rotational transformations (Monti et al., 2017; Fawaz et al., 2021), it lacks formal equivariance guarantees. Future versions of GeoMorph will investigate the integration of theoretically grounded rotationally equivariant convolution frameworks, such as SE(3)-equivariant networks (Fuchs et al., 2020). However, thorough evaluation and additional development will be required, as these networks typically impose a significant computational complexity burden.

Section 4.2 presents compelling evidence of the advantages offered by GeoMorphAll in achieving enhanced registration outcomes. The inclusion of myelin and rfMRI features in the registration process has proven to be beneficial for individual-to-template alignment. A comparison between GeoMorphAll and MSMAll results, as demonstrated in Figs. 7 and 8 using both HCP and UKB datasets, reveals that GeoMorphAll produces a sharper and clearer average map in the UKB dataset when compared to MSMAll. This difference may arise from the fact that MSMAll was originally optimized for the HCP dataset and that the feature extraction network in GeoMorph is more advantageous in the context of the UKB dataset, where the data is considerably noisier compared to the HCP dataset.

The computational complexity of GeoMorph is heavily dependent on the resolution of the control points. As a result, we have been limited to a control grid on an icosphere of level 4 due to memory constraints. Hence, future extensions of this work will explore more efficient methods to overcome this limitation. Additionally, while the CRF has proven to be beneficial in regularizing the deformation field, there is a keen interest in investigating other regularization techniques that fully leverage the characteristics of the problem. One promising approach is the development of a mechanically learnable regularization penalty that considers the physical properties of brain tissues. This approach aims to obtain improved and robust deformations while also allowing for topology-breaking transformations. Furthermore, it is worth noting that GeoMorph’s implementation is entirely based on MoNet convolutions that somewhat limit the expressivity of the features derived from the network (Fawaz et al., 2021). Improved performance might be achievable through use of fully expressive, rotation-equivariant surface convolutions such as Cohen et al. (2018) and Wiersma et al. (2020). Alternatively, the incorporation of surface transformer networks, leveraging the attention mechanism as proposed by Dahan et al. (2022), could also lead to improvements by enabling more precise feature alignment through enhanced context awareness and better handling of complex surface variations.

## Figures and Tables

**Table 1:** Distortions measures and average runtime for different methods at CC ~ 0.88. Classical methods (top) and learning-based methods (bottom).

Methods	CC similarity	P-value	Areal distortion					Shape distortion					Avg. time	
			Mean	Max	95%	98%		Mean	Max	95%	98%		CPU	GPU
Freesurfer	0.75	0.324	0.34	11.73	0.82	1.00		0.63	6.77	1.29	1.54		30 min	–
MSM Pair	0.877	0.170	0.41	9.17	1.24	1.76		0.62	9.05	1.61	2.16		13 min	–
MSM Strain	0.880	0.165	0.27	**1.06**	0.53	0.66		0.64	**1.93**	1.17	1.30		1 h	–
SD	0.875	0.172	**0.18**	2.00	**0.50**	**0.65**		**0.24**	1.98	**0.50**	**0.65**		**1 min**	–
S3Reg	0.875	0.162	0.26	22.22	0.82	1.16		0.51	21.65	1.35	2.0		8.8 s	8.0 s
GeoMorph	0.875	0.174	**0.19**	**2.43**	**0.53**	**0.69**		**0.26**	**2.70**	**0.63**	**0.82**		**8.3 s**	**2.6 s**

**Table 2:** Multimodal registration results: CC, distortions measures, and average runtime for different methods. HCP (top) and UKB (bottom).

Methods	CC similarity			Cluster mass			Areal distortion						Shape distortion						Avg. time	
	Myelin	rfMRI		Myelin	rfMRI		Mean	Max		95%	98%		Mean	Max		95%	98%		CPU	GPU
MSMSulc	0.938	0.523		5269	130 800		0.1	0.35		0.16	0.18		0.21	0.64		0.38	0.42		40 min	–
GeoMorphSulc	0.954	0.523		5382	133 575		0.19	2.43		0.53	0.69		0.26	2.70		0.63	0.82		8.3 s	2.6 s
MSMAll	0.945	0.566		5546	182 636		0.27	1.2		0.62	0.68		0.62	1.92		1.13	1.24		1.5 h	–
GeoMorphAll	**0.975**	**0.569**		**5797**	**182734**		0.24	7.57		0.64	0.84		0.35	8.5		0.82	1.0		**7.7 s**	**0.55 s**
MSMSulc	0.75	0.35		96 476	71 105		0.34	0.1		0.35	0.17		0.23	0.65		0.41	0.45		40 min	–
GeoMorphSulc	0.94	0.36		96591	80 973		0.19	2.43		0.52	0.68		0.30	3.20		0.68	0.87		8.3 s	2.6 s
MSMAll	0.944	**0.40**		96 779	**105 866**		0.27	2.4		0.63	0.70		0.66	3.10		1.2	1.40		1.5 h	–
GeoMorphAll	**0.96**	**0.40**		**97 319**	103 358		0.28	7.72		0.86	1.21		0.41	8.78		1.0	1.53		**7.7 s**	**0.55 s**

**Table 3:** Cluster mass estimates of the aligned HCP tfMRI data using proposed methods.

Task name	MSMSulc	GeoMorphSulc	MSMAll	GeoMorphAll
Emotion	251 677	248 641	**303 403**	290 406
Gambling	352831	350 187	**418681**	405 669
Relational Processing	550403	552 769	**611565**	602 924
Language Story	558 434	570 587	634 654	**654 618**
Social Cognition	625291	618 237	687 310	**690586**
Motor	1485 991	1487 278	**1617727**	1612465
Working Memory	1961 989	1936 564	**2240 070**	2190214

**Table 4:** Results of tuning control point and labels grid resolution.

Performance at multiple control point resolutions.			
Measure	ico-2	ico-3	ico-4
CC Similarity: Myelin	0.969	0.972	**0.975**
CC Similarity: rfMRI	0.563	0.568	**0.569**
Areal Distortion: 95%	**0.55**	0.60	0.64
CM: Myelin	5657	5778	**5797**
CM: rfMRI	175967	182 510	**182734**
CM: Language Story	641 751	649 699	**654618**
CM: Motor	1591248	1605 115	**1612 465**
Performance with labels on an icosphere of order 5.			
Measure	Value		
CC Similarity: Myelin	0.973		
CC Similarity: rfMRI	0.567		
Areal Distortion: mean	0.24		
Areal Distortion: max	8.30		
Areal Distortion: 95%	0.65		
Shape Distortion: mean	0.34		
Shape Distortion: max	8.8		
Shape Distortion: 95%	0.83		

**Table 5:** Ablation experiments using HCP data.

CRF-RNN	CC similarity			Areal distortion					Shape distortion			
	Myelin	rfMRI		Mean	Max	95%	98%		Mean	Max	95%	98%
GeoMorphAll full model performance (based on Table 2).												
✓	0.975	0.569		**0.24**	**7.57**	**0.64**	**0.84**		**0.35**	**8.5**	**0.82**	**1.0**
GeoMorphAll registration results without CRF-RNN regularization network.												
✗	0.976	0.569		0.26	8.64	0.71	0.91		0.39	9.4	0.86	1.32
Results of the deformation learned as a velocity field with scaling and squaring layers.												
NA	0.967	0.554		0.34	10.04	1.0	1.36		0.49	9.6	1.23	1.64
Comparisons between GeoMorph (top) and DDR (bottom) using HCP sulcal depth feature.												
✓		0.875		0.19	2.43	0.53	0.69		0.26	2.70	0.63	0.82
✓		0.875		0.19	2.66	0.53	0.71		0.26	3.14	0.66	0.86

**Table 6:** GeoMorphAll results on UKB.

Training data	CC similarity			Areal distortion					Shape distortion			
	Myelin	rfMRI		Mean	Max	95%	98%		Mean	Max	95%	98%
HCP + UKB	**0.96**	**0.40**		0.28	**7.72**	0.86	1.21		0.41	**8.78**	1.0	1.53
HCP	0.955	0.395		**0.20**	10.11	**0.56**	**0.76**		**0.30**	10.21	**0.70**	**0.91**

## Data Availability

The authors do not have permission to share data.

## Footnotes

2
https://github.com/ThomasYeoLab/CBIG

3 Available through FSLv6.0.

4
https://github.com/ecr05/MSM_HOCR

5
https://github.com/zhaofenqiang/SphericalUNetPackage
